# [^68^Ga]Ga-Pentixafor and Sodium [^18^F]Fluoride PET Can Non-Invasively Identify and Monitor the Dynamics of Orthodontic Tooth Movement in Mouse Model

**DOI:** 10.3390/cells11192949

**Published:** 2022-09-21

**Authors:** Rogerio B. Craveiro, Alexandru Florea, Christian Niederau, Sihem Brenji, Fabian Kiessling, Sabri E. M. Sahnoun, Agnieszka Morgenroth, Felix M. Mottaghy, Michael Wolf

**Affiliations:** 1 Department of Orthodontics, University Hospital RWTH Aachen, 52074 Aachen, Germany; 2 Department of Nuclear Medicine, University Hospital RWTH Aachen, 52074 Aachen, Germany; 3 Department of Radiology and Nuclear Medicine, Academic Hospital Maastricht, 6229 HX Maastricht, The Netherlands; 4 School for Cardiovascular Diseases (CARIM), Maastricht University, 6229 ER Maastricht, The Netherlands; 5 Institute for Experimental Molecular Imaging, University Clinic Aachen, RWTH Aachen University, 52074 Aachen, Germany

**Keywords:** calcification, [^68^Ga]Ga-Pentixafor, inflammation, in vivo remodeling, long-term monitoring, orthodontic tooth movement, PDL, periodontal ligament, Sodium [^18^F]Fluoride (Na[^18^F]F)

## Abstract

The cellular and molecular mechanisms of orthodontic tooth movement (OTM) are not yet fully understood, partly due to the lack of dynamical datasets within the same subject. Inflammation and calcification are two main processes during OTM. Given the high sensitivity and specificity of [^68^Ga]Ga-Pentixafor and Sodium [^18^F]Fluoride (Na[^18^F]F) for inflammation and calcification, respectively, the aim of this study is to assess their ability to identify and monitor the dynamics of OTM in an established mouse model. To monitor the processes during OTM in real time, animals were scanned using a small animal PET/CT during week 1, 3, and 5 post-implantation, with [^68^Ga]Ga-Pentixafor and Na[^18^F]F. Both tracers showed an increased uptake in the region of interest compared to the control. For [^68^Ga]Ga-Pentixafor, an increased uptake was observed within the 5-week trial, suggesting the continuous presence of inflammatory markers. Na[^18^F]F showed an increased uptake during the trial, indicating an intensification of bone remodelling. Interim and end-of-experiment histological assessments visualised increased amounts of chemokine receptor CXCR4 and TRAP-positive cells in the periodontal ligament on the compression side. This approach establishes the first in vivo model for periodontal remodelling during OTM, which efficiently detects and monitors the intricate dynamics of periodontal ligament.

## 1. Introduction

The orthodontic tooth movement (OTM) treatment is the alignment of displaced teeth, eliminating malocclusions and inadequate loading of the periodontium. The periodontium is essential for supporting the functionality of the tooth, composed of diverse of mineralized and non-mineralized tissues such as cementum, periodontal ligament (PDL) and alveolar bone [1]. OTM is a process initiated by sterile inflammation and orthodontic force which induces periodontal bone remodeling [2]. The sterile inflammation is a reaction to mechanical stimulation where the local tissue induces an inflammatory response (i.e., synthesis of pro-inflammatory mediators including enzymes, cytokines and chemokines) in order to initiate local tissue remodeling [3,4,5,6]. During OTM, the teeth are loaded with a specific mechanical force whose vector points in the direction of the desired tooth movement [7]. This mechanical force triggers stress/strain distribution in the periodontal ligament (PDL), causing local hypoxia and fluid flow and initiating a sterile inflammatory cascade enabling bone remodeling. PDL fibroblasts play a unique and dominant role in the regulation of bone remodeling during OTM [8]. Bone resorption occurs on the compression side with resorption of the alveolar bone and degradation of the periodontal ligament, while new bone is formed on the tension side with stretching of the periodontal ligament inducing bone apposition and alignment of the Sharpey fibers [9,10,11,12]. Although there are various animal models in orthodontic research trying to explain molecular and cellular mechanisms of OTM, no longitudinal animal studies with continuous periodontal remodeling using in vivo monitoring have been performed so far.

To assess the dynamics of orthodontic tooth movement in an established preclinical model, this study therefore aims at identifying and in vivo monitoring the inflammatory process inducing periodontal tissue remodeling. The innovative approach of monitoring the periodontal remodeling in real time enables one to follow the OTM in a complex and dynamic way throughout all its phases.

From a clinical point of view, OTM occurs in three phases. The first phase is the initial damping, where the tooth is deflected within the alveolar cavity by the width of the periodontal gap and is compressing the periodontal ligament. It is followed by a second phase (i.e., hyalinization phase) in which nearly no tooth movement is detected. The third phase (i.e., resorption phase) is reached when an accelerated and homogeneous tooth movement can be recognized due to direct bone resorption [13].

Sodium [^18^F]Fluoride (Na[^18^F]F) position emission tomography (PET) represents an emerging modality with the potential for early diagnosis and monitoring of active bone remodeling through the detection of subtle metabolic changes [14,15,16]. Na[^18^F]F is a PET tracer that specifically binds to hydroxyapatite [17]. Small nano-sized hydroxyapatite crystals found during (periodontal) bone remodeling provide an ideal target for Na[^18^F]F, in contrast to regular bone, which contains large crystals that are hard to penetrate [18]. For this reason, it is thought that Na[^18^F]F is an adequate tracer to image active bone remodeling [18]. Specifically, the dissociated Fluoride-18 is incorporated into hydroxyapatite, and its uptake reflects osteoblastic activity, allowing for the quantification of bone turnover [14]. [^68^Ga]Ga-Pentixafor has recently been introduced for imaging of the chemokine receptor CXCR4, a receptor involved in the hematopoiesis and inflammation. CXCR4 is specifically co-localized with monocytes/macrophage infiltration [19] and inflammatory diseases of the bone [20] and plays a role in immune cell migration during atherosclerosis progression [21].

Based on this knowledge, the scope of this study is to assess the feasibility of dynamically monitored inflammation and periodontal bone remodeling using non-invasive positron emission tomography/computed tomography (PET/CT).

With two imaging probes, Na[^18^F]F and [^68^Ga]Ga-Pentixafor, addressing inflammation and calcification respectively, it is possible to assess their ability to identify and non-invasively monitor the dynamics of orthodontic tooth movement in an established mouse model. This dynamic imaging of periodontal remodeling will contribute to understanding the in vivo intricate dynamics of the periodontal ligament inflammation and bone remodeling and will also enable a long-time prospective observation of the same animal or subject.

## 2. Materials and Methods

### 2.1. Study Design and Experimental Animals

A total of 12 male C57BL/6JRj wild-type mice (WT) (age: 10–11 weeks, Janvier Labs, Le Genest-Saint-Isle, France) were used to induce experimental OTM. All animals received a Nickel-Titan coil spring for anterior movement of the first upper left molar. The contralateral untreated jaw served as a non-force internal control (split-mouth model) and all measurements were performed on both jaw sides. For the small animal PET/CT, 9 mice (*n* = 9) per time point (1, 3, and 5 weeks) were examined. After the in vivo analysis at weeks 1, 3 and 5, two animals were finalized according to legal guidelines for histology evaluation. In order to avoid unnecessary suffering of the animals, corresponding in vivo tracer administration and termination criteria were predefined, and animal condition as well as body weight were monitored. Before inserting the orthodontic appliance, the mice were fed with standard rodent chow feed and had access to water ad libitum in plastic bottles. One week before the implantation of stretched closed-coil spring, the mice received soft food AIN 93 water soluble (SSNIF GmbH, Soest, Germany) with gelatin for the acclimatization phase. After the insertion of the stretched closed coil, the mice received additionally DietGel Recovery (Clear H_2_O, Westbrook, ME, USA).

### 2.2. Orthodontic Tooth Movement

**Figure 1:**
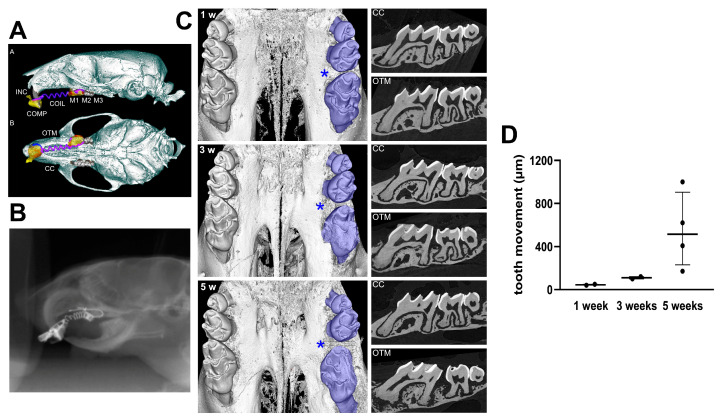
Periodontal remodelling and orthodontic tooth movement after 1, 3, and 5 weeks with a NITi coil spring compared to the untreated contralateral jaw side. (**A**) Orthodontic apparatus employed in mouse maxilla, upper jaw. Schematic representation based on micro-computed tomography scans showing 3-dimensional sagittal and occlusal views of an orthodontic appliance used in mouse. A super elastic coil spring (COIL) was fixed between the maxillary left first molar (M1) and maxillary incisors (INC) using a dental composite (COMP) to achieve mesial (anterior) movement. The loaded side is labelled as the orthodontic tooth movement (OTM) side, while the contralateral control (CC) side received no orthodontic appliance. (**B**) Imaging of mouse under isoflurane anaesthesia during CT scans. (**C**) Three-dimensional and two-dimensional micro-CT reconstruction of mouse maxillary molars under OTM or CC sides. Displacement of the first maxillary molar (MI) from contact with the second molar (M2) is indicated by asterisk. Occlusal view displaying both OTM and CC sides, with asterisks indicating greater separation between MI and M2 in WT mice. (**D**) Mesial tooth movement in µm after NiTi coil spring activation of the first molar during weeks 1, 3, and 5.

For insertion of the NiTi coil spring, animals were positioned on their back in a custom-made surgical bed. The upper jaw was immobilized by an anatomical probe spanning across the palate between the upper incisors and molars, which was connected to the bed via filaments, thus stretching the head down in order to enable easy access to the oral cavity. This was performed according to a modified method described previously for rats [22,23,24] and described in our previous works [25]. Briefly, the mice were isoflurane-anesthetized, and a NiTi coil spring with a force level of 0.25N was inserted. The appliance consisted of a stretched closed-coil spring (0.012-inch nickel-titanium wire, Dentaline GmbH) ligated between the maxillary left first molar and maxillary incisors using dental composite restoration. A split-mouth design employed right maxillary first molar as a contra-lateral control (CC) (Figure 1). The appliance delivered the force in the mesial/anterior direction, causing mesial tipping of the first molar. After the experimental period of 5 weeks, the animals were finalised.

### 2.3. Tracer Synthesis

One Na[^18^F]F synthesis was performed for each scanning day. The quaternary methyl ammonium (i.e., QMA) carbonate cartridges (186004540, Waters GmbH, Eschborn, Germany) were first conditioned with a 0.9% NaCl solution then washed with sterile water. Afterwards, crude [^18^F]Fluoride (proton-irradiated target water) was loaded onto the cartridge, washed with sterile water, and eluted with a 0.9% NaCl. This was used for the intravenous injections.

One [^68^Ga]Ga-Pentixafor synthesis was also performed for each scanning day. [^68^Ga]Ga-Pentixafor was produced by a routine procedure primarily used for patient care. For this purpose, a cassette synthesizer type GRP 3 V (Scintomics, Fürstenfeldbruck, Germany) was used with SC-01 cassettes (ABX, Radeberg, Germany) using HEPES buffer during labeling. Briefly, 10 mL of ^68^GaCl_3_ containing 0.6 M HCl generator eluate (iThemba, Somerset West, South Africa) was diluted with water and trapped on a cation exchange SPE cartridge, eluted with 5 M NaCl, and added to the reactor containing 50 µL of an aqueous (0.25 mg/mL) DOTA functionalized Pentixafor derivative (Scintomics Molecular ATT, Fürstenfeldbruck, Germany) and HEPES. After labeling reaction (120 °C, 10 min), HEPES was removed by reversed phase SPE extraction. The product was eluted from a C8 cartridge by 0.1 mL aqueous EtOH (80%) followed by formulation with 0.9 mL PBS. Radiochemical purities were >98%.

### 2.4. Small Animal PET/CT Acquisition, Reconstruction and Quantification

All mice were imaged with a small animal PET/SPECT/CT system (i.e., Tri-umph^®^ II, Northridge Tri-Modality Imaging, Inc., Chatsworth, CA, USA); however, only the PET and CT modalities were used for this study.

Under 1.5–2.5% isoflurane anesthesia in oxygen at 0.8 L/min, 10 ± 2 MBq of Na[^18^F]F in a maximum total volume of 125 μL was injected in the lateral tail vein. After injection, the mice were placed on the scanner bed and the CT scan was initiated. The exposure settings used were as follows: 130 uA, 75 kVp, 230 ms exposure time, and 360° rotation with 720 views with an average of two frames for each view; the duration of the CT scans was ~15 min. PET scan (duration 1 h) was initiated at the end of the CT scan (i.e., approximative 25 min post injection). The CT had an axial field of view of 59.2 mm and the PET had one of 112 mm. During the scans, the isoflurane concentration was adapted to achieve a respiratory rate between 75–50 breaths per minute.

On the following day, each animal underwent the same procedure, however, after injection of 10 ± 2 MBq of [^68^Ga]Ga-Pentixafor, the performed PET scan duration was only 45 min.

CT images were reconstructed using a Feldkamp filtered back projection reconstruction process to a voxel size of 0.154 × 0.154 × 0.154 mm^3^ in a 592 × 592 × 560 matrix. Using vendor software, the CT values were converted into Hounsfield units (HU) using the formula HU = 1000 × ((µ_t_ − µ_w_)/µ_w_) where µ_w_ is the linear attenuation coefficient of the water and µ_t_ is the linear attenuation coefficient of the tissue.

The PET data were reconstructed using a 3D ordered-subset expectation maximization (i.e., OSEM-3D with three iterations and eight subsets) with a maximum a posteriori probability algorithm (30 iterations) into a 240 × 240 × 192 image matrix (resulting in final voxel dimensions of 0.25 × 0.25 × 0.597 mm^3^). PET normalization, CT attenuation correction, and CT scatter correction were applied to all PET reconstructions.

PET images were automatically aligned to the CT using a custom-made transformation in PMOD software package version 3.13 (PMOD Technologies LLC, Zürich, Switzerland) from a capillary phantom. The co-registered PET/CT images were further used for quantification.

For both tracers first the right upper molar region (i.e., including the first molar) was manually masked and the 50th percentile of the generated volume of interest was recorded as a background activity for the scan. A similar volume of interest was afterwards manually drawn around the left upper molar region. Finally, an automatic isocontour was generated using the recorded background activity as the minimal threshold for both left and right volumes of interest, and their total uptake was recorded.

To quantify the PET data and to correct for the blood compartment contribution, a target-to-background ratio (TBR) was calculated using the following formula: TBR = Total_target_/Total_background_, where Total_target_ is the total tracer activity of the left (i.e., OTM side) region and Total_background_ is total tracer activity of the right region (i.e., internal control).

### 2.5. Ex vivo High-Resolution CT Analysis

Maxilla of the mice were scanned using a high-resolution micro-CT scanner (Skyscan 1272, Bruker, Belgium 60 kVp, 166 µA, 424 ms integration time). Briefly, they were dissected, deskeletonized, and split into two equal sagittal parts. The left side of the jaw was stored in 70% EtOH for µCT evaluation. The images consisted of an isometric voxel size of 3 µm. Two-dimensional images were used to generate 3D reconstructions using 3D visualization software (ImalyticsPreclinical v2.1, Aachen, Germany). The 3D reconstruction was used to evaluate the orthodontic tooth movement.

### 2.6. Preparation of Paraffin Sections

At the end of the 1st week, 3rd week, and at the last PET/CT scan in the 5th week, animals were finalized by decapitation under deep isoflurane anesthesia, the upper jaw was collected and fixed in 3.7% paraformaldehyde solution for at least 24 h. The samples were transferred to ethanol 70% until the CT measurements. Samples were split into OTM and CC sides. Afterwards, they were decalcified with 10% Tris-buffered ethylene diamine tetra-acetic solution (EDTA, pH 7.4) (Morphisto, Offenbach, Germany) at room temperature for 8 weeks. The buffer was renewed every 2 days. Once decalcified, each sample was embedded in paraffin, and sectioned for histologic analyses. Serial sagittal sections of the upper jaw 5 µm thick were prepared for TRAP staining and IHC for CXCR4.

### 2.7. Histology

Paraffin-embedded sagittal sections of the maxillae were incubated overnight at 37 °C and then hydrogenated via descendent alcohol series and rinsed in distillate water. Two micrographs of molar were randomly selected from each animal for histological analysis. The stained histological sections were digitized using a Zeiss Observer 7 microscope (Zeiss, Jena, Germany).

### 2.8. Tartrate-Resistant Acid Phosphatase (TRAP) Staining

To verify findings in Na[^18^F]F uptake, a tartrate-resistant acid phosphatase (TRAP) staining was performed to identify osteoclast/odontoclast-like cells [26]. A leukocyte acid phosphatase (TRAP) kit was used according to the manufacturer’s instructions (387A Sigma-Aldrich, Steinheim, Germany). Subsequently, the sections were covered with Aquatex (1.08562.0050, Merck, Darmstadt, Germany). To quantify the number of TRAP-positive multinucleated cells, the first and second root in the compression zone were photographed and measured (TRAP-positive cells per 1 mm) using Zen software (version 3.3 Zeiss, Jena, Germany).

### 2.9. CXCR4 Immunohistochemical Staining

To confirm respective findings in [^68^Ga]Ga-Pentixafor uptake, immunohistochemistry of CXCR4 was performed on paraffin-embedded sections. Antigen retrieval was performed using proteinase K (E00492, Life Technologies, Vilnius, Lithuania). For peroxidase blocking, slides were incubated in 0.3% H_2_O_2_. Samples were blocked with blocking horse serum (H0146, Sigma-Aldrich). Tissues were then incubated with a primary monoclonal rabbit (UMB2) anti-CXCR4 antibody (5 µg/mL, 124824, abcam ab, Berlin, Germany) at 4 °C overnight. Then, sections were incubated with a secondary goat anti-rabbit HRP-antibody (1111-035-003, Dianova, Eching, Germany) and with 3.3-diaminobenzindine (DAB) (K346811-2, Dako, Jena, Germany) for visualization. The sample was counter-stained with hematoxylin (Roth T865.1). Subsequently, tissue slides were washed in running tap water. Finally, all slides were mounted with Aquatex (1.08562.0050, Merck, Darmstadt, Germany) for microscope imaging.

### 2.10. Statistical Analysis

All statistical analyses were performed using GraphPad Prism version 9 (GraphPad Software LLC, San Diego, CA, USA). Initially, all experimental datasets (i.e., divided into 1st, 3rd, and 5th week per tracer) were tested for outliers using the ROUT method. To determine a statistical difference between each data set and a TBR theoretical mean of 1.0, a one-sample *t*-test was performed. This test determines whether there is any difference between the sample values and the theoretical mean of 1.0, which represents the TBR value if there is no difference between the left- and right-side uptake.

To calculate a statistical difference between multiple groups, non-parametric one-way analysis of variance (i.e., Kruskal–Wallis test) tests were applied. Additionally, the standard deviation of all variables was calculated and used alongside the average in the Results section. If the differences from this test exceeded the statistical significance threshold (i.e., *p* < 0.05), Dunn’s correction for multiple comparisons was performed for a post hoc analysis. Statistically significant results are indicated in charts, with asterisks suggesting a *p* value lower than 0.05.

All variables are presented as data dots with a line indicating the mean and error bars for the standard deviation.

For histology analysis Mann–Whitney U-tests were performed (Prism version 9.0.0; GraphPad Software, Dr. Harvey Motulsky, San Diego, CA, USA) where *p <* 0.05 was considered statistically significant. The graphs show the individual data values as well as the median values 95% confidence interval.

## 3. Results

### 3.1. Long Time Orthodontic Tooth Movement Appliance

The effect of in situ orthodontic appliance of NiTi coil spring was analyzed for a long time, over the period of 5 weeks, using a non-invasive live in vivo procedure. (Figure 1A–C) At the respective time points (weeks 1, 3, and 5), the mesial orthodontic tooth movement was 45 ± 7 µm (week 1), 155 ± 7 µm (week 3), and 545 ± 348 µm (week 5) (Figure 1D).

### 3.2. Na[^18^F]F Has an Increased Uptake in the Region under OTM

**Figure 2:**
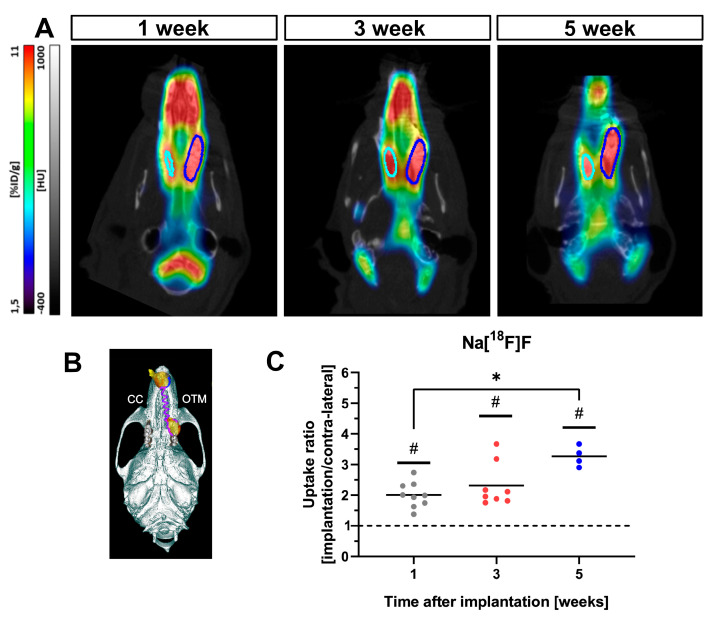
Na[^18^F]F longitudinal uptake. (**A**) Na[^18^F]F uptake increases in the region of interest (left superior molar region, under orthodontic tooth movement) compared to the contralateral side, which was taken as a reference for background uptake. The deep-blue contour represents the region of interest taken for the OTM side, while the turquoise one represents the contralateral side of the jaw, which was taken as a control. (**B**) OTM and control (CC) sides as revealed by the 3D rendering of the CT scan. (**C**) Significant differences compared to 1 were visible in all scanning weeks, with significant difference between the 1st and the 5th week as well. *: statistically significant difference compared to 1st week, #: statistically significant difference compared to 1.0.

Na[^18^F]F-PET detected an increased uptake in the region under orthodontic tooth movement compared to the contralateral side, an internal reference for the background uptake. Moreover, the Na[^18^F]F uptake increased in intensity over study time (target-to-background ratio was 2 ± 0.43 in week 1, 2.3 ± 0.7 in week 3, and 3.2 ± 0.3 in week 5), indicating an acceleration of bone remodelling after NiTi coil spring implantation (Figure 2).

### 3.3. [^68^Ga]-Pentixafor Has an Increased Uptake in Early and Late Phase of OTM

**Figure 3:**
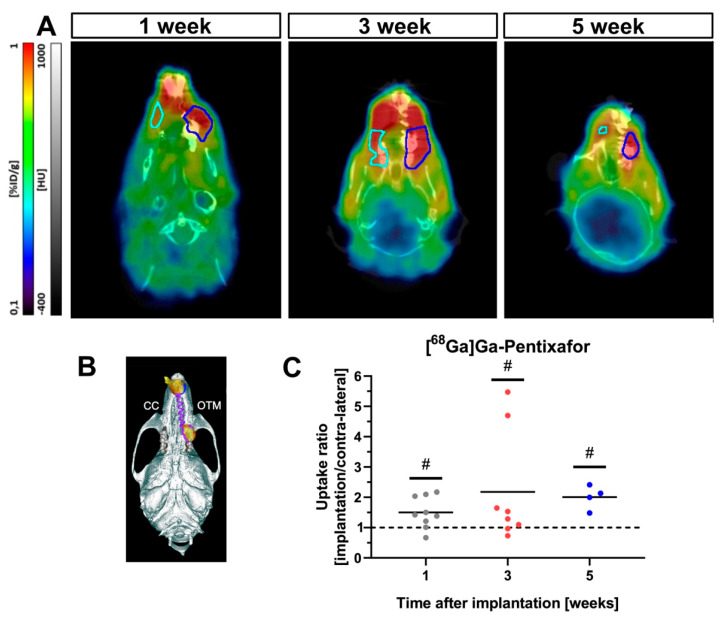
[^68^Ga]Ga-Pentixafor uptake increases in the early and late phase of OTM. (**A**) A constant uptake of [^68^Ga]Ga-Pentixafor was observed during the 5-week trial, suggesting a persisting presence of inflammation. The deep-blue contour represents the region of interest taken for the OTM side, while the turquoise one represents the contralateral side of the jaw, which was taken as a control. (**B**) OTM and control (CC) sides as revealed by the 3D rendering of the CT scan. (**C**) Significant differences of the uptake ratio were compared to 1 in the 1st and 5th weeks; the 3rd week also showed a significant difference compared to 1 as a result of the two animals with a TBR > 4. There are no differences of the TBR between the scanning weeks, suggesting a constantly increased uptake. #: statistically significant difference compared to 1.0.

PET scans visualized an increased uptake in the OTM region compared to the contra-lateral side in the 1st, 3rd, and 5th week. A constant uptake of [^68^Ga]Ga-Pentixafor was observed during the 5-week trial after NiTi coil spring implantation (target-to-background ratio was 1.5 ± 0.5 in week 1, 2.1 ± 1.8 in week 3, and 2.0 ± 0.3 in week 5), suggesting a permanent presence of inflammatory markers (Figure 3).

### 3.4. Increased Presence of CXCR4-Positive Cells in OTM Side Compared to the Untreated Contralateral Jaw Side

**Figure 4:**
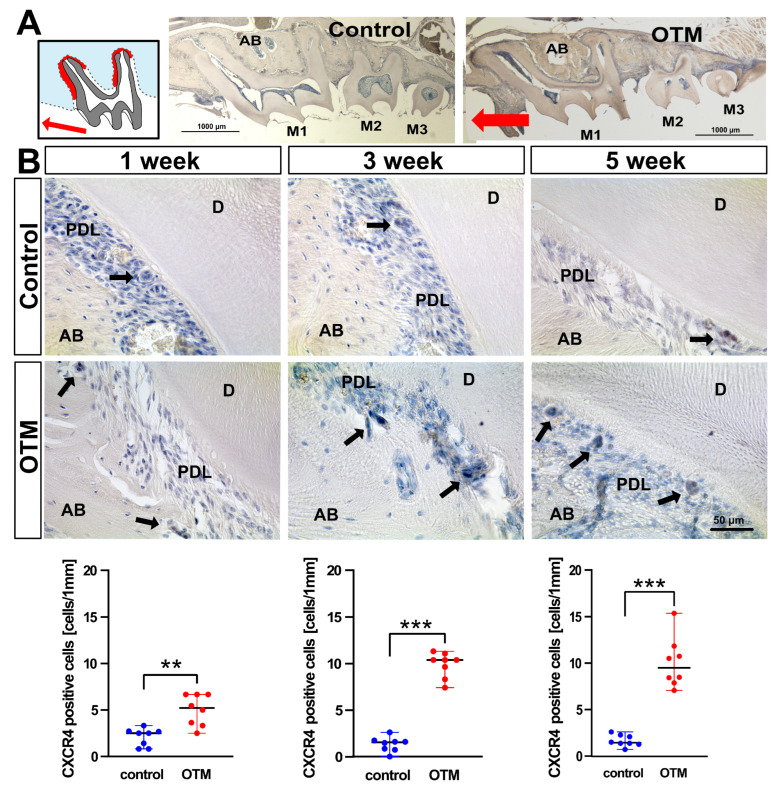
Increase in CXCR4-positive cells at the OTM side with NiTi coil spring compared to the untreated contralateral jaw side. (**A**) Schematic picture of compression zones in M1 distal roots (red) and overview pictures of sagittal sections of maxillary mice, contralateral control side and orthodontic tooth movement (OTM) side. (**B**) Focusing on the compression zones of M1 distal roots, a number of CXCR4-positive cells (black arrows) were counted on the OTM side and CC. On CC sides the baseline number of CRCR4-positive cells is not significantly different, while on the OTM side it exhibits a significant increase at all time points (** *p* < 0.01 and *** *p* < 0.001, respectively). Two micrographs of molars were randomly selected from at least two animals for histological analysis.

CXCR4-positive cells were quantified on compression zones of M1 distal roots on OTM with the contralateral jaw side (CC) as a control. Compared to the CC, a significant increase of CXCR4-positive cells on periodontal ligament in OTM side with a NiTi coil spring was detected at all time points (1 week, *p* = 0.0023; 3 weeks, *p* < 0.0002; and 5 weeks, *p* = 0.0002) (Figure 4).

### 3.5. Osteoclastogenesis Increases in All OTM Phases

**Figure 5:**
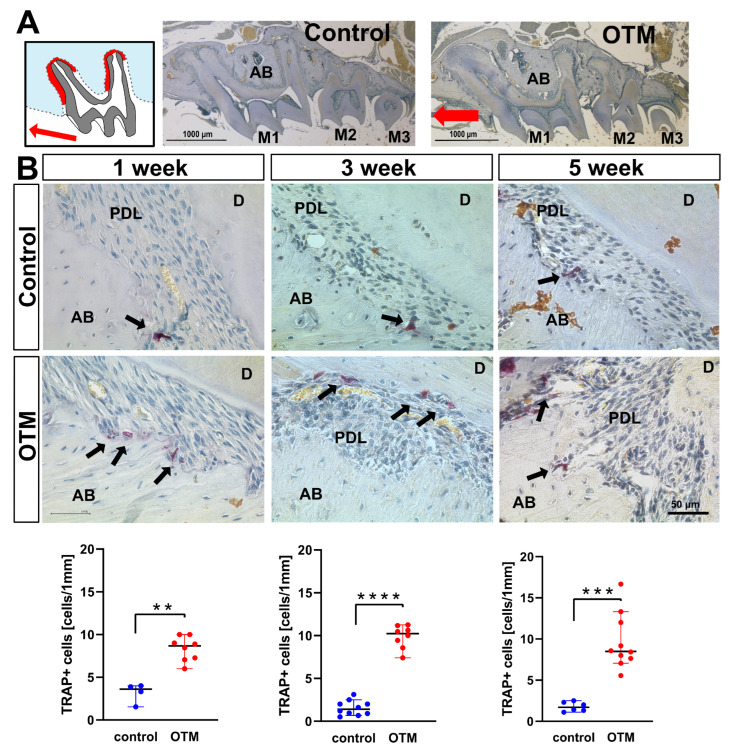
Increase of TRAP-positive cells corresponding to osteoclastogenesis at the OTM side with NiTi coil spring compared to the untreated contralateral jaw side. (**A**) Schematic picture of compression zones’ M1 distal roots (red) and overview pictures of sagittal sections of maxillary mice, contralateral control side and orthodontic tooth movement (OTM) side. (**B**) Focusing on the compression zones of M1 distal roots, the number of TRAP-positive osteoclast/odontoclast-like cells (black arrows) was counted on the OTM side and CC. On CC sides the baseline number of TRAP-positive cells is not significantly different, while on the OTM side, it exhibits a significant increase at all time points (** *p* < 0.01, **** *p* < 0.0001, and *** *p* < 0.001, respectively). Two micrographs of molars were randomly selected from at least two animals for histological analysis.

TRAP-positive cells were quantified on compression zones of M1 distal roots at the OTM and CC side, the latter serving as a control. TRAP-positive cells were detected predominantly at the alveolar bone (AB) side, with nearly none of them located at the cementum side. Compared with the contralateral jaw side (CC), OTM with a NiTi coil spring significantly increased the number of TRAP-positive cells at all time points (1 week, *p*= 0.004; 3 weeks, *p* < 0.0001; and 5 weeks, *p* = 0.0002) (Figure 5).

## 4. Discussion

Despite increasing clinical interventions aimed at shortening treatment time [27], the average active orthodontic treatment takes 18–24 months, which is a lengthy commitment [12,28]. New approaches enabling one to monitor the molecular mechanism of orthodontic force-induced periodontal remodelling longitudinally can therefore valuably contribute to the needs of clinical practice. Until now, only short-time studies with a maximum of 3 weeks OTM in mice were performed with relevant evaluation [29,30,31,32,33,34]. Our study presents for the first time an innovative approach to follow the complexity and dynamics of OTM over a long time (5 weeks) with an in vivo monitoring of the inflammatory process inducing periodontal tissue remodelling.

According to previous studies, the NiTi coil spring method is the recommended method for long-term experiment [35]. However, the insertion of NiTi coil spring is time consuming and technically demanding with a higher risk of injury during insertion accompanied with an acute loss of body weight immediately after implantation due to discomfort in the oral cavity, leading to a high mortality rate of animals in the first days. Nevertheless, it is the only method to achieve long-term fixation. NiTi coil spring enables a more constant tooth movement within longer experimental periods. In our study, the method resulted as being in line with previous reports [25,35] on a bodily movement, in which the tooth as a whole is changed in its position and tooth tipping, here anterior tipping of the moved first molar. Such OTM can lead to bone remodelling and tooth position stability over time. From a molecular point of view, the jaw remodelling is a coordinated tissue resorption and formation in the tooth surrounding bone and periodontal ligament. Under healthy body conditions, osteoclast and osteoblast homeostasis maintains the integrity of the skeletal system. During orthodontic treatment, the cellular activities of osteoclasts and osteoblasts become abnormal.

Based on this biological process during OTM, [^68^Ga]Ga-Pentixafor and Sodium [^18^F]Fluoride are accurate tracers to investigate and visualise the dynamics of OTM in vivo. As already shown, [^68^Ga]Ga-Pentixafor [19,36,37,38,39,40] and Na[^18^F]F [41,42] are useful for the early diagnosis and monitoring of certain inflammatory and bone diseases, respectively [14]. As presented in this study, the sterile inflammation induced during OTM [13] is assessable by [^68^Ga]Ga-Pentixafor, a ligand for CXCR4. This receptor is specifically co-localized with monocytes/macrophages that are involved in bone infiltration [19] during inflammatory diseases [20]. This result is supported by new findings with AMD3100, an antagonist of CXCR4 receptor. The local administration of AMD3100 was shown to control initial OTM and to diminish the bone resorption processes during OTM via inhibition of the SDF-1/CXCR4 axis. [43] In our study, [^68^Ga]Ga-Pentixafor has a good TBR in the first and 5th week, suggesting its potential for detection and visualization in the initial and post-lag phase, but not in the lag phase (i.e., 3rd week) of OTM. At this time point, 25% of the investigated animals still showed a TBR > 4, suggesting the potential of [^68^Ga]Ga-Pentixafor for the investigation of remodeling processes in the lag phase of OTM. However, further studies are required to reveal the reason behind the higher uptake in these animals. CXCR4 was described as an osteoblast marker that has effects on the mesenchymal stem cell pool and on allocation to the osteoblastic and adipocytic cell lineages [44]. Besides, it was also shown that CXCR4 was also expressed in pre-osteoclasts and enhanced in mature osteoclasts. Specifically, LPS does not influence the expression of SDF-1/CXCR4 in osteoblasts, but up-regulates the expression of CXCR4 in pre-osteoclasts via Toll-like receptor 4, which subsequently enhances pre-osteoclast migration [45]. Moreover, SDF-1/CXCR4 signaling in the mature osteoblasts can feedback to regulate the osteoclast precursor pool size and play a multifunctional role in regulating bone formation and resorption [44]. These findings corroborate with the significant increase of CXCR4-positive cells in periodontal ligament on the compression zones of M1 distal roots during OTM as well as with [^68^Ga]Ga-Pentixafor uptake.

Sodium [^18^F]Fluoride is a PET tracer that specifically binds to hydroxyapatite [17], by which it can reveal the direct role of osteoblast activity during bone remodelling processes. Moreover, due to its specificity, Na[^18^F]F can visualize lytic bone changes that are accompanied by a component of abnormal osteoblast activity [46].

Na[^18^F]F showed time dependently increasing TBR, which suggests its potential for accurate identification and monitoring of OTM. The increase in the Na[^18^F]F uptake with time enables detection in both early and later stages of OTM.

During the last decade, it has become clear that PDL fibroblasts contribute to the in vitro differentiation of osteoclasts and adapt to bacterial and mechanical stimuli by synthesizing higher levels of osteoclastogenesis-stimulating molecules [47,48]. In our previous studies [49,50,51], we demonstrated in line with many others [52,53,54,55] that an immunomodulatory potential of human PDL cells via the release of mediators modifies the macrophages polarization and differentiation as a function of periodontal repair during OTM. Especially on the compressive site, macrophages participate in inflammatory processes’ mediation through proinflammatory cytokines, induction of neoformation and angiogenesis of blood vessel formation factors and extracellular matrix proteins’ remodeling [56]. Here we demonstrate that in a long-term OTM, the increase of CXCR4-positive and TRAP-positive cells correlated with [^68^Ga]Ga-Pentixafor and Na[^18^F]F uptake, respectively. Thus, [^68^Ga]Ga-Pentixafor as well as Na[^18^F]F PET are useful methods for a direct non-invasive in vivo monitoring of inflammation and bone remodeling during OTM.

## 5. Conclusions

This study proved the feasibility of in vivo non-invasive monitoring of orthodontic tooth movement using a combination of radioactive tracers and molecular imaging techniques already used in the clinical routine. Since Na[^18^F]F and [^68^Ga]Ga-Pentixafor are already approved for clinical use, our results have validated the translational value of both as diagnostic tool for OTM restricted to animal models. Live molecular imaging enables the investigation of animal models with various periodontal diseases or knockout models. Thus, this approach significantly contributes to understanding the intricate dynamics of periodontal ligament inflammation, calcification, and bone remodelling. This valuable knowledge will enhance the efficient OTM modulation and treatment of periodontal diseases in patients.

## Data Availability

The data that support the findings of this study are available from the corresponding author, R.B.C., upon reasonable request.
